# Langerhans Cell Modulation in Atopic Dermatitis Is TLR2/SOCS1‐Dependent and JAK Inhibitor‐Sensitive

**DOI:** 10.1111/all.16641

**Published:** 2025-07-09

**Authors:** Yuxuan Deng, Nicole Leib, Sylvia Schnautz, Said Benfadal, Johannes Oldenburg, Thomas Bieber, Nadine Herrmann

**Affiliations:** ^1^ Center for Skin Diseases University Hospital Bonn Bonn Germany; ^2^ Christine Kühne‐Center for Allergy Research and Education (CK‐CARE) Davos Switzerland; ^3^ Institute of Experimental Hematology and Transfusion Medicine University Hospital Bonn Bonn Germany; ^4^ Department of Dermatology University Hospital Zürich Zürich Switzerland; ^5^ Bieber Dermatology Consulting Bonn Germany

**Keywords:** atopic dermatitis, JAK/STAT, Langerhans cells, microbiome, TLR2

## Abstract

**Background:**

Langerhans cells (LC) are epidermal dendritic cells building the skin's outermost immunological barrier and bridging innate and adaptive immune responses. Their sensing property of the microbiome via Toll‐like receptors (TLR) is impaired in atopic dermatitis (AD). We hypothesize a desensitization of LC because of persistent *Staphylococcus aureus* exposure in AD and underlying mechanisms being TLR2‐related.

**Methods:**

Human LC generated from hematopoetic stem cells were desensitized via repetitive exposure to TLR2‐ligands (priming) and compared to unprimed cells for their TLR‐responsiveness. JAK inhibitors impact was evaluated. Maturation marker, migration marker and behavior, cytokine release, and downstream molecule regulation were addressed by flow cytometry, qPCR, and transwell and multiplex assays.

**Results:**

Primed LC mimicked the LC behavior in AD skin, exhibiting desensitization toward TLR2‐mediated activation monitored by impaired CD83/CD80/CD86 and MHCII expression as well as impaired regulation of chemokines CCR6 and CCR7, migration competence, and Th17‐driving cytokines. IL‐18 and IL‐1β were elevated under these conditions. Negative regulators of the TLR2 pathway, specifically SOCS1 and IRAKM, were significantly upregulated, whereas activating molecules were hardly affected. JAK inhibitors reduced SOCS1 expression in primed cells and restored activation markers CD83/80/86 and MHCII upon TLR2 engagement, but had no effect on IRAKM expression.

**Conclusion:**

Primed LC mimic the impaired LC‐responsiveness toward TLR2 in AD skin. Our findings unravel a new direct contribution of LC to AD‐associated IL‐1β and IL‐18 under these conditions and shed light on the mechanistical role of SOCS1 and the mode of action of JAK inhibitors.

## Graphical abstract

Constitutive exposure to TLR2 ligands can modify Langerhans cell (LC) activation capacity and outcome, being similar to LC in atopic dermatitis skin. While maturation marker and migration are impaired, the cytokine profile switches and favors atopic dermatitis‐related IL‐1β and IL‐18. This modification is mechanistically mediated by increase of inhibitory molecules SOCS1 and IRAK‐M and sensitive to JAK inhibitors. CCR, chemokine receptor; CD, cluster of differentiation; IKK, IκB kinase; IL, interleukin; IRAK, interleukin‐1 receptor‐associated kinase; JAK, Janus kinase; LC, Langerhans cell; MHC, major histocompatibility complex; NEMO, NF‐κB essential modulator; NF‐κB, nuclear factor‐kappa B; Pam3Cys, TLR2 ligand; SOCS, suppressor of cytokine signaling; TAB, TGF‐β activated kinase 1 binding protein; TAK, TGF‐beta activated kinase; TLR, Toll‐like receptor; TNF, tumor necrosis factor; TRAF, tumor necrosis factor receptor‐associated factor. Partly created in BioRender. Deng, Y. and Herrmann, N. (2025) https://BioRender.com/inoli8s.

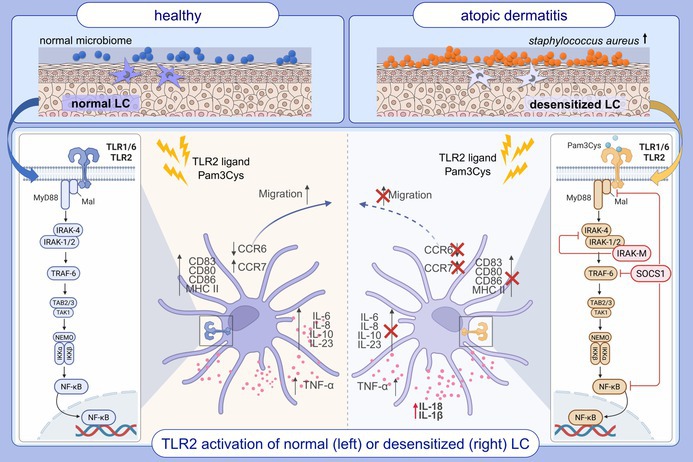

**Abbreviations**
AD: Atopic dermatitis
APC: antigen‐presenting cells
CCR6: chemokine CC receptor 6
CCR7: chemokine CC receptor 7
DC: Dendritic cells
ILC2: type 2 innate lymphoid cells
JAK: Janus kinase
LC: Langerhans cells
rFI: relative Fluorescence Index

*S. aureus*

:
*Staphylococcus aureus*

TLR: Toll‐like receptors

## 1 Introduction

Atopic dermatitis (AD) is the most common chronic inflammatory skin disease, with a high prevalence in children, and because of its itchy character and visible lesions, it places a high burden on the patients and patients families quality of life. Additionally, AD can introduce other atopic diseases like food allergies, allergic rhinitis, or asthma, being the starting point of a distinct immunological journey of the individual [1, 2]. The interaction of a variety of factors, like epidermal barrier dysfunction, immune dysregulation, genetic and environmental aspects, and increased vulnerability to infections, makes AD a complex and heterogenic disease [3, 4, 5]. Immunologically, AD is mainly characterized as a type‐2 inflammation with high levels of Th2 and ILC2‐driven IL‐4/IL‐13, IL‐5, and IL‐9, IgE‐producing B cells, and high levels of serum IgE [6]. However, increasing evidence for different subtypes manifests with the lack of a general “one‐fits‐all” drug and subsequent need for individualized therapies, as well as dissected analyses and characterization of AD patient endotypes, mirroring the disease's complexity.

Langerhans cells (LC), as the predominant subtype of dendritic cells (DC) in the epidermis and superficial skin layers [7], play a crucial role in maintaining skin homeostasis and tolerance [8, 9]. Their presence in the skin at the outermost layer allows them to effectively survey and respond to environmental stimuli [10]. Because of their antigen‐presenting character, LC can sense pathogens via pattern recognition receptors such as Toll‐like receptors (TLR), and subsequently activated LC can migrate to the draining lymph nodes and present antigens to T cells, initiating their immune response and polarization, particularly Th17 cell responses against bacteria [9, 11, 12]. This classical view on LC as a dendritic cell subtype has expanded during the last years with the recognition of macrophage characteristics of these cells and their unique dual identity [9]. The detailed mechanisms regulating the functional preference of LC are still not fully understood, but their multiple abilities may mirror their importance at the border between innate and adaptive immune responses.

The significance of resident LC in the pathogenesis of AD has been increasingly recognized. The skin microbiome in AD, especially in the lesions, is dysregulated and shows an increased general bacterial load with *Staphylococcus aureus* ( *S. aureus* ). LC are capable of sensing and responding to *S. aureus* via TLR2. The interaction between LC and *S. aureus* is essential in mounting an effective immune response against this pathogen [12, 13, 14].

TLR play a crucial role in sensing both extracellular and intracellular pathogens [15]. In AD, several TLR, including TLR1, TLR2, TLR6, TLR3, TLR4, and TLR9, and their influence on inflammation, as well as pruritus and the skin barrier, have been described [16, 17, 18]. Among the TLR family members, TLR2 stands out for its wide pathogen recognition, triggering immune responses upon activation. Its activation initiates a cascade of immune events in response to pathogen invasion [19]. The TLR2‐NF‐κB signaling pathway contributes to the activation of DC and LC, their cytokine release, and the subsequent T cell response [19]. Upon pathogen recognition, TLR2 engages MyD88, leading to IRAK family member complex formation (IRAK1, IRAK2, and IRAK4). This activates TRAF6 and TAK, driving NF‐κB translocation to the nucleus. NF‐κB then serves as a transcription factor involved in various biological processes [20]. This intricate signaling pathway orchestrates the immune response to pathogens, highlighting the importance of TLR2 in innate immunity [21]. The signaling cascade, in turn, is regulated by inhibitory molecules like SOCS, IRAKM, A20, and others. In AD patients, there is a downregulation of TLR2 expression, leading to dysregulated activation and impaired function of TLR2 [9, 22]. This also contributes to the disruption of tight junction proteins and antimicrobial peptides, which are crucial for maintaining a healthy skin barrier [9].

In our previous study, we demonstrated that LC from AD ex vivo skin exhibited a significantly impaired response to TLR2‐mediated classical activation [22]. In contrast, LC from the skin of anti‐inflammatory‐treated AD patients were robust in their TLR2 response [23]. However, the underlying mechanisms responsible for this desensitization of LC and their role in the pathogenesis of AD remain unclear.

In inflammation, cytokines play a pivotal role as they maintain and define the inflammatory characteristics [24, 25]. Binding to their receptors results in the activation of Janus kinases (JAK) and the initiation of the JAK/STAT signaling pathway. Different cytokine receptors recruit different JAK family members, like JAK1, JAK2, JAK3, or TYK2, to mediate the downstream signaling. JAK inhibitors have evolved as interesting anti‐inflammatory therapeutics in multiple diseases [26, 27]. They inhibit the kinase activity of JAK by blocking the ATP binding site or modulating elements within the enzyme, resulting in a blockage of the signaling cascade and subsequent suppression of the inflammation. JAK inhibitors can act more generally or can be selective for certain JAK. In AD, JAK inhibitors are therapeutic agents and interfere with, for example, IL‐4/IL‐13 signaling, TSLP, IL‐5, and IL‐31 signaling, the generation of inflammatory dendritic epidermal cells (IDEC) and have multiple inflammatory modes of action [28, 29, 30, 31].

We hypothesized that the impaired reaction to TLR2 ligands in AD skin is a result of increased *S. aureus* abundance. To address this issue, we (i) established a mimicking in vitro system using CD34+ hematopoietic stem cells derived LC [32], (ii) analyzed and confirmed the desensitization of LC and analyzed its underlying mechanisms in the TLR2‐NF‐κB pathway, (iii) showed that LC under these conditions can serve as a source of AD‐related cytokines, thus contributing to AD immune dysregulation, and (iv) showed evidence supporting a new putative mode of action of JAK inhibitors within the context of impaired TLR engagement in LC in AD.

## 2 Methods

### 2.1 Cells

Cells were obtained from human umbilical cord blood from voluntary donors at St. Mary's Hospital, Bonn, or buffy coats from voluntary blood donors at the Institute of Experimental Hematology and Transfusion Medicine, University Hospital Bonn. The study was ethically approved by the University of Bonn's local ethics committee (no. 204/09) and conducted following the Declaration of Helsinki principles [33], with informed consent from all participants. All samples were anonymized and all experiments were conducted in vitro. No donor information was considered.

### 2.2 Generation, Priming, and Stimulation of LC

**FIGURE 1:**
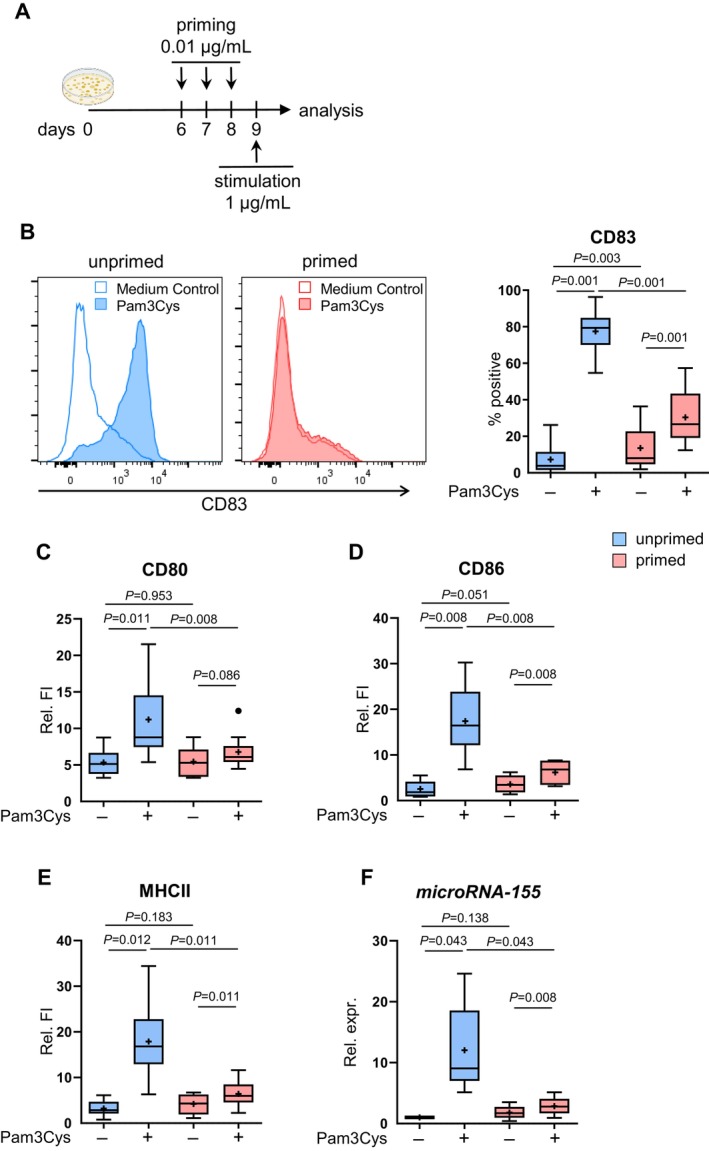
Primed LC are impaired in TLR2‐mediated upregulation of CD83, CD80/86, MHCII, and microRNA‐155. Primed LC were generated by repeated treatment with 0.01 μg/mL of Pam3Cys on days 6, 7, and 8 (A). Unprimed (blue) and primed cells (red) were left unstimulated (−) or stimulated with 1 μg/mL Pam3Cys (+) for 24 h (B–E) or 6 h (F). Cells were stained with CD1a and CD14 for gating CD34LC as described previously, 7‐AAD for the exclusion of dead cells, and CD83 (B, *n* = 20), CD80 (C, *n* = 9), CD86 (D, *n* = 9), MHCII (E, *n* = 9), or isotype control and analyzed by flow cytometry. Boxplots show % of positive cells (A) or relative fluorescence intensity (Relative FI; B–D). The histograms show one representative experiment with unprimed (left) and primed (right) cells after Pam3Cys stimulation or without stimulation (empty). CD1a cells were enriched via magnetic separation and subjected to Taqman qPCR for microRNA‐155 expression (F, *n* = 5) and relative expression fold change after normalization to RUN48 is presented. Results are presented as boxplots, with the mean as “+” and outliers as “**•**”. Statistical significance was assessed using SPSS, applying the paired Wilcoxon signed rank test. *P*‐values are shown above the boxes.

LC were generated from CD34+ hematopoietic stem cells isolated from cord blood or buffy coat by immunomagnetic beads (Miltenyi Biotec, Bergisch Gladbach, Germany), as described previously [32]. Viable CD34+ hematopoietic stem cells with a purity of > 85%, analyzed by flow cytometry with 7‐AAD and CD34 antibodies or isotype control, were cultured in RPMI supplemented with 10% FCS, 1% antibiotics/antimycotics, 10 μM 2‐mercaptoethanol, and in the presence of GM‐CSF (300 U/mL), TNF‐α (20 U/mL), stem cell factor (10 ng/mL), FLT‐3 L (10 ng/mL), and TGF‐β (0.5 ng/mL). On day 4 of the culture, ½ the volume of the culture media was exchanged with fresh media containing GM‐CSF and TGF‐β [32]. For primed LC, cells were additionally treated with 0.01 μg/mL Pam3Cys or 1.0 ng/mL LPS every 24 h for three cycles starting at day 6 or 7 (Figure 1A). Unprimed control cells were treated with equal volumes of media accordingly. 24 h after the third priming, cells were stimulated with 1.0 μg/mL Pam3Cys, 100 ng/mL LPS, or media only for unstimulated control for the indicated time and analyzed by flow cytometry, transwell assay, qPCR, and cytokine release (described in the Supporting Information).

### 2.3 JAK Inhibitor Treatment

On day 8, cells were treated with JAK inhibitors with different selectivities (ruxolitinib (JAK1/2), filgotinib (JAK1), BMS‐911543 (JAK2), and decernotinib (JAK3), InvivoChem, Vernon Hills, IL). The IC50 values are shown in Table S3. A concentration of 1 μmol/L was chosen on the basis of previous studies for selective JAK inhibitor while maintaining cell viability [30] and DMSO as a control. Subsequently, cells were stimulated with Pam3Cys.

### 2.4 Analyses

Statistical analyses were conducted using SPSS 24 (IBM Deutschland GmbH, Ehningen, Germany) with the paired Wilcoxon signed rank test. n is the number of individual experiments, each of which was set from one blood source. *p* < 0.05 was considered significant. Graphs were created with GraphPad Prism 9 (GraphPad Software, San Diego, USA), and the abstract graph was generated using BioRender (www.biorender.com).

## 3 Results

### 3.1 Exposure to Low Doses of TLR2 Ligand Pam3Cys Results in Impaired TLR2‐Driven Activation in LC

In the first approach, we generated hematopoietic stem cell‐derived LC and mimicked the constitutive exposure to *S. aureus* in atopic skin by repetitive exposure to low doses of TLR2 ligand Pam3Cys [22]. We analyzed the resulting cells (primed LC) in their capacity to react to TLR2 stimulation with a high dose of TLR2 ligand, as described previously [32], and compared it to cells without priming (unprimed LC) (Figure 1A).

Firstly, the priming procedure itself slightly induced CD83 but did not activate LC, indicated by remarkably lower maturation marker CD83 than Pam3Cys‐activated LC, and similar expression of co‐stimulatory markers CD80, CD86, and MHC II in unstimulated primed cells compared to unstimulated unprimed cells (Figure 1B–E). However, robust TLR2‐driven LC maturation was evident by upregulated CD83, CD80, and CD86, and MHC II after Pam3Cys stimulation in unprimed cells (Figure 1B–E), whereas in primed LC, the upregulation of these markers was remarkably impaired (Figure 1B–E) for at least 96 h (Figure S1). Furthermore, microRNA‐155, involved in DC maturation and regulated by TLR ligands, was significantly elevated in unprimed LC after Pam3Cys stimulation, in contrast to stimulated primed cells (Figure 1F).

In summary, low‐dose Pam3Cys priming effectively mimicked an impaired responsiveness of LC to TLR2‐driven maturation in vitro, as described for LC in AD skin.

### 3.2 Desensitization of LC Occurred With Both Homologous Priming and Activation via TLR2 or TLR4 as Well as Heterologous TLR2/4 Priming and Activation

Next, we investigated the priming of LC in the context of different TLR ligands. LC were primed with Pam3Cys (TLR2 ligand) or LPS (TLR4 ligand) and then exposed to the same ligand (homologous activation) or a different TLR ligand (heterologous activation).

**FIGURE 2:**
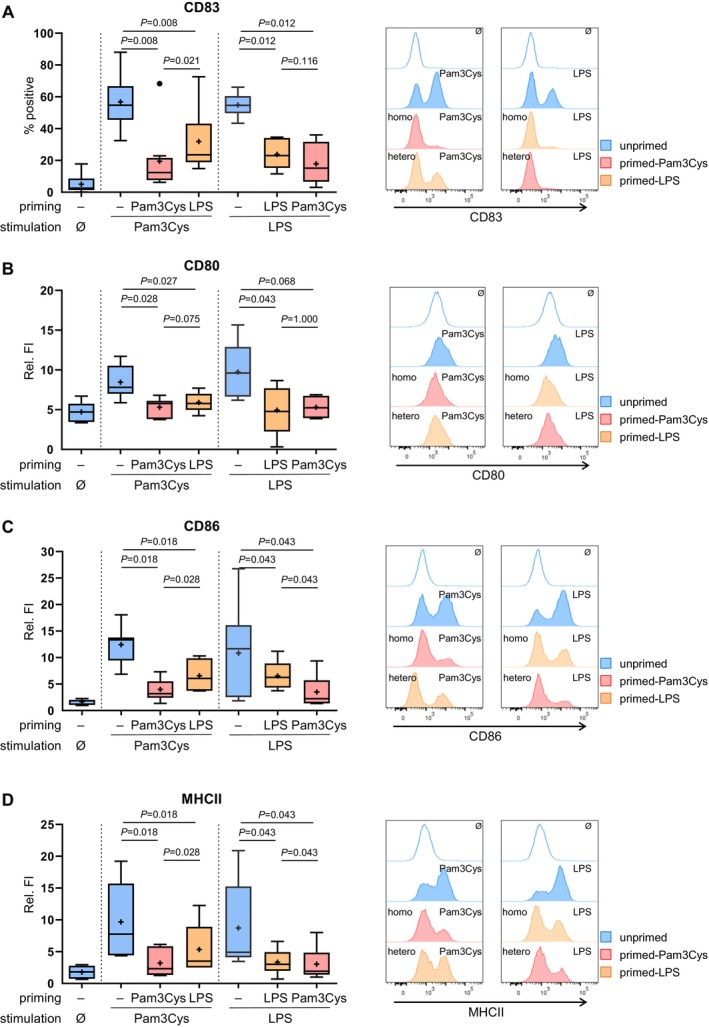
Maturation of LC is impaired after TLR2 or TLR4 priming followed by both heterologous and homologous stimulation. LC were cultured for 6 days and subjected to either unprimed or primed conditions using the TLR2 ligand Pam3Cys (0.01 μg/mL) or the TLR4 ligand LPS (1 ng/mL), every 24 h for three cycles. Unprimed, Pam3Cys‐primed cells, and LPS‐primed cells were exposed to Pam3Cys (1 μg/mL) or LPS (100 ng/mL) for 24 h, or left unstimulated (−) and subjected to flow cytometry. Cells were stained with CD1a and CD14, 7‐AAD for the exclusion of dead cells, and CD83 (A, *n* = 9), CD80 (B, *n* = 6), CD86 (C, *n* = 7), MHCII (D, *n* = 6), or isotype control and analyzed by flow cytometry. The histograms show one representative experiment depicting unprimed (blue), primed‐Pam3Cys (red), and primed‐LPS (orange) after Pam3Cys or LPS stimulation or medium control (empty). Boxplots show % of positive cells (A) or relative fluorescence intensity (Relative FI; B‐D) with the mean as “+” and outliers as “**•**”. Statistical significance was assessed using SPSS, applying the paired Wilcoxon signed rank test. *P*‐values are shown above the boxes.

LPS triggered LC activation, as shown by significant CD83, CD80, CD86, and MHC II upregulation in unprimed cells (Figure 2), whereas LPS‐primed cells exhibited low expression of these markers upon LPS stimulation, indicating TLR4‐driven LC desensitization. Notably, Pam3Cys‐primed cells exhibited a reduced induction of CD83, CD80, CD86, and MHC II following stimulation with LPS compared to unprimed cells (Figure 2A–D). Similar outcomes were observed in LPS‐primed cells stimulated with Pam3Cys. These results show a desensitization of LC to TLR ligands after both homologous and heterologous TLR2 and TLR4 priming.

Furthermore, we observed a higher expression of CD83, CD86, and MHC‐II in LPS‐primed cells than in Pam3Cys‐primed cells upon Pam3Cys stimulation, whereas no significant difference between them was observed upon LPS stimulation (Figure 2A–D). This shows that TLR2‐activation discriminates between homologous and heterologous priming, whereas TLR4‐activation is less selective on the priming agent.

In conclusion, our findings suggested that LC desensitization to TLR ligands follows both homologous TLR2 activation and heterologous TLR4 activation, whereas exhibiting distinct responses to TLR2 and TLR4‐driven stimulations.

### 3.3 Primed LC Exhibit Impaired Migratory Competence Upon TLR2‐Engagement

**FIGURE 3:**
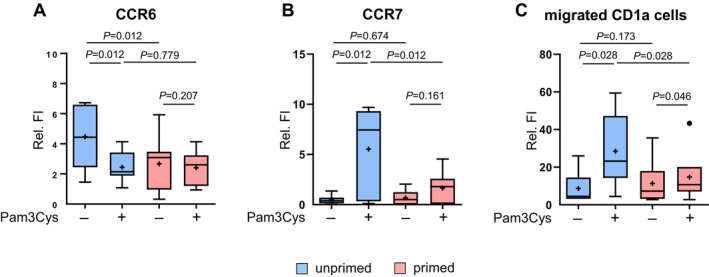
Priming induces impaired migratory capacity after TLR2 stimulation. LC were generated and subjected to either primed conditions with 0.01 μg/mL Pam3Cys or unprimed conditions. Subsequently, cells were stimulated with Pam3Cys (1 μg/mL) for 24 h or left unstimulated (−). (A‐B) Cells were stained with CD1a and CD14, 7‐AAD for the exclusion of dead cells and CCR6 (A, *n* = 8), CCR7 (B, *n* = 8) or isotype control and analyzed by flow cytometry. Boxplots present the relative fluorescence intensity (Relative FI). (C) 1 × 10^6^ cells of each condition were resuspended in 100 μL media and transferred to a transwell chambers, and then incubated for 6 h. Migrated cells were counted and analyzed for CD1a expression by flow cytometry. The migrated CD1a cells are presented as the percentage of all CD1a cells subjected to the assay (*n* = 6, Cell number %). Results are presented as boxplots, with the mean as “+” and outliers as “**•**”. Statistical significance was assessed using SPSS, applying the paired Wilcoxon signed rank test. *P*‐values are shown above the boxes.

Consistent with previous studies, the migratory competence of LC induced by TLR2 activation was found to be impaired in AD^22^. Therefore, we further investigated whether the priming treatment also affected the migration of LC in vitro. Firstly, we analyzed the expression levels of chemokine CC receptor 6 (CCR6), which is expressed on immature LC and is involved in LC recruitment and maintenance in the tissue [34], and chemokine CC receptor 7 (CCR7), which promotes migration to lymph nodes and is usually expressed on activated cells [35]. Unprimed LC showed a significant decrease in CCR6 expression and an increase in CCR7 expression after Pam3Cys stimulation, indicating TLR2‐induced migration activation (Figure 3A,B). In primed LC, CCR6 was expressed at a lower level and its downregulation in response to Pam3Cys was lacking (Figure 3A). The stimulation‐induced upregulation of CCR7 was also impaired in primed LC (Figure 3B).

Next, we applied a transwell assay and analyzed the migration behavior against the chemoattractant CCL19, which is a ligand of CCR7. Unprimed LC showed an elevated number of migrated cells (Figure 3C), confirming that TLR2‐driven activation induces LC migration against CCL19. However, the number of migrated cells in primed cells only slightly increased upon subsequent Pam3Cys stimulation, and the number was significantly lower than in unprimed cells.

These findings reveal impaired TLR2‐induced migratory competence in desensitized LC, characterized by diminished regulation of chemokine receptors and reduced migration, accompanied by a general lower CCR6 expression.

### 3.4 Distinct Cytokine Profiles of Primed LC Upon TLR2 Activation

Next, we investigated the cytokine profile of primed LC in response to TLR2‐driven activation. For this purpose, cytokine production in the supernatant after Pam3Cys stimulation of both unprimed and primed LC was analyzed.

**FIGURE 4:**
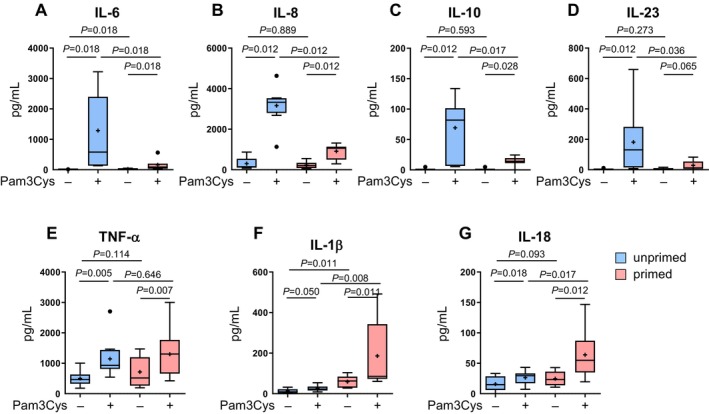
Primed LC show a distinct cytokine profile after TLR2 stimulation with elevated IL‐18 and IL‐1β. Pam3Cys‐primed and unprimed LC were stimulated with 1 μg/mL Pam3Cys and IL‐6 (A, *n* = 7), IL‐8 (B, *n* = 8), IL‐10 (C, *n* = 7), IL‐23 (D, *n* = 8), TNF‐α (E, *n* = 10), IL‐1β (F, *n* = 9), and IL‐18 (G, *n* = 8) concentrations in culture supernatants were assessed using LEGENDplex Cytometric Bead Assay (BioLegend), with each sample analyzed in triplicate. Results are presented as boxplots, with the mean as “+” and outliers as “**•**”. Statistical significance was assessed using SPSS, applying the paired Wilcoxon signed rank test. *P*‐values are shown above the boxes.

Consistent with previous studies [36, 37] engagement of TLR2 induced the release of pro‐inflammatory cytokines IL‐6, IL‐8, IL‐23, TNF‐α, and immunomodulatory cytokine IL‐10 in unprimed LC (Figure 4A–E). However, primed LC showed a reduced induction of IL‐6, IL‐8, IL‐23, and IL‐10 after Pam3Cys stimulation (Figure 4A–D). Notably, the pro‐inflammatory cytokine TNF‐α was hardly affected by the priming treatment (Figure 4E).

Surprisingly, primed LC exhibited substantial IL‐1β and IL‐18 induction upon Pam3Cys stimulation, whereas unprimed cells had minimal induction (Figure 4F,G). Moreover, priming alone also slightly but significantly elevated IL‐1β production.

Taken together, primed LC displayed a distinct cytokine profile upon TLR2‐driven activation, marked by decreased pro‐inflammatory cytokines, IL‐6, IL‐8, IL‐23, and IL‐10, and significantly elevated AD‐related cytokines IL‐1β and IL‐18.

### 3.5 Regulatory Molecules SOCS1 and IRAKM of the TLR2‐NF‐kB Signaling Pathway Mediate the Priming Induced LC Desensitization

To unravel underlying mechanisms, we focused on the TLR2‐NF‐κB pathway. TLR2 ligand engagement activates a series of downstream molecules including MyD88, IRAK1, IRAK2, IRAK4, and TRAF6, leading to the transcription of series genes [21]. We analyzed the expression profile of these activatory molecules by qPCR in primed versus unprimed LC.

**FIGURE 5:**
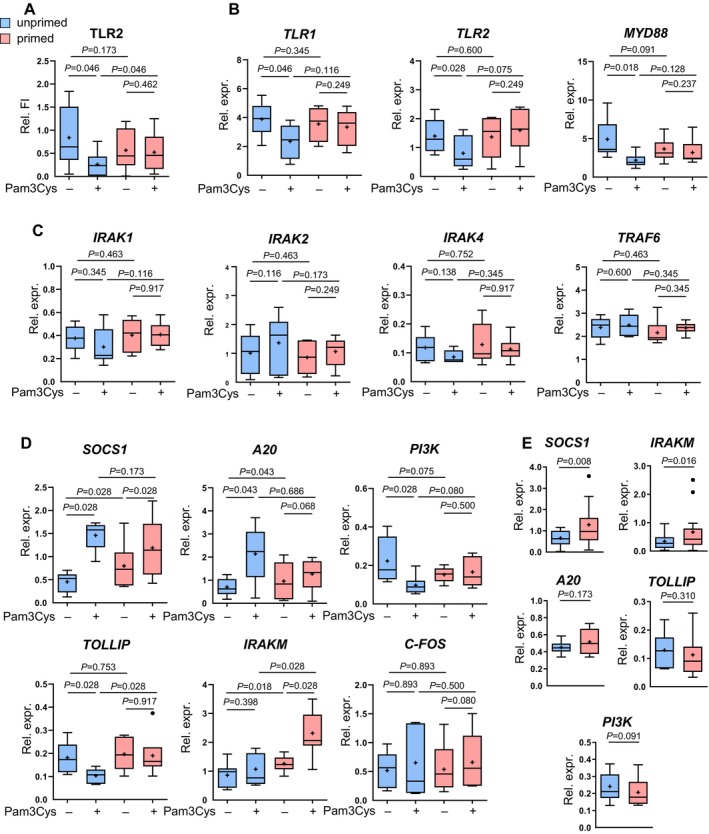
Negative regulators SOCS1 and IRAKM, but not activatory molecules of the TLR2‐NF‐κB signaling pathway, are affected in primed LC. Pam3Cys‐primed and unprimed LC were left unstimulated (−) or stimulated with 1 μg/mL Pam3Cys (+) for 24 h (A) or 6 h (B–D). (A) Cells were stained with CD1a and CD14 for gating LC as described previously, 7‐AAD for the exclusion of dead cells, and TLR2 (*n* = 6) or isotype control and analyzed by flow cytometry. Boxplots show relative fluorescence intensity (Relative FI). (B–D) CD1a cells were enriched via magnetic separation and subjected to qPCR for mRNA expression for*TLR1* (*n* = 6), *TLR2* (*n* = 6), *MYD88* (*n* = 7), *IRAK1* (*n* = 6), *IRAK2* (*n* = 6), *IRAK4* (*n* = 6), *TRAF6* (*n* = 6), *SOCS1* (*n* = 7), *A20* (*n* = 6), *PI3K* (*n* = 6), *TOLLIP* (*n* = 6), *IRAKM* (*n* = 7), and *C‐FOS* (*n* = 5). (E) Pam3Cys‐primed and unprimed LC were analyzed for *SOCS1* (*n* = 12), *IRAKM* (*n* = 13), *A20* (*n* = 6), *TOLLIP* (*n* = 6), and *PI3K* (*n* = 6) by qPCR 24 h after the last priming treatment. Boxplots show relative expression fold change after normalization to housekeeping gene. Results are presented as boxplots, with the mean as “+” and outliers as “•”. Statistical significance was assessed using SPSS, applying the paired Wilcoxon signed rank test. *P*‐values are shown above the boxes.

The expression of TLR2 decreased with TLR2 engagement on the cell surface as well as on the mRNA level, suggesting a transcriptional regulation of TLR2 by its stimulation in unprimed cells (Figure 5A,B, blue boxes). This regulation is absent in primed cells, underlining the inhibitory character of the priming treatment. TLR2 surface expression was lower in primed than in unprimed LC (Figure 5A); however, the mRNA expression of TLR1/2 and other activatory molecules showed no significant differences on the basis of the priming treatment itself (Figure 5B,C, left blue box vs. left red box). The activation of the LC was independent of the TLR2 surface variety, as unprimed LC with low or high TLR2 expression monitored by a similar CD83 increase after Pam3Cys stimulation (Figure S2, blue circles). Similarly, the lack of activation in primed cells was observed independent from TLR2 expression (Figure S2, red triangles). TLR1/2 and MyD88 mRNA in unprimed LC were downregulated after TLR2 stimulation. However, primed LC lacked TLR2 and MyD88 regulation, confirming that priming impaired TLR2 activation. No significant alterations were observed in IRAK1, IRAK2, IRAK4, and TRAF6 expression levels in neither primed nor unprimed LC after Pam3Cys stimulation (Figure 5C). However, a trend of IRAK1 and IRAK4 downregulation and IRAK2 upregulation after TLR2 stimulation was observed in unprimed cells, but not in primed cells. Taken together, these results, on the one hand, confirm that the priming treatment impairs TLR2 response in LC. On the other hand, the results do not suggest a transcriptional regulation of the TLR2‐NF‐κB pathway, though the regulation of surface TLR2 may be mechanistically involved.

Next, we investigated whether a negative feedback mechanism is induced by the priming treatment. We addressed expression levels of several intracellular negative regulators of the TLR signaling pathway, namely, TOLLIP, A20, IRAKM, PI3K, and SOCS1 [38, 39].

In unprimed LC, Pam3Cys stimulation upregulated SOCS1 and A20, downregulated TOLLIP and PI3K, whereas IRAKM expression remained unchanged (Figure 5D). However, in primed cells, the regulation of A20, TOLLIP, and PI3K was absent, indicating the impaired reaction toward TLR stimulation. Conversely, SOCS1, A20, and IRAKM exhibited upregulation by the priming alone and were further elevated upon Pam3Cys stimulation (Figure 5D). We confirmed that SOCS1 and IRAKM were already significantly elevated after the priming at the time point of stimulation (Figure 5E), whereas activation marker CD83, CD80, CD86, and MHCII remained at low levels (Figure S3), indicating that priming itself keeps the immature phenotype of LC independent of SOCS1 and IRAKM expression.

Taken together, this provides insights into the mechanisms by which LC desensitization toward TLR activation occurs and is associated with the negative feedback molecules SOCS1 and IRAKM.

### 3.6 JAK Inhibitors Restore the Maturation of Primed LC to TLR2‐Driven Activation and Reduce IL‐18 and IL‐1β

Next, we studied JAK inhibitors as potent AD drugs known to alleviate AD symptoms [40] on primed LC activated by TLR2 ligation. Primed LC were treated with ruxolitinib (JAK1/2), filgotinib (JAK1), BMS‐911543 (JAK2), and decernotinib (JAK3) for 12 h and then exposed to Pam3Cys and the phenotype and maturation was analyzed.

**FIGURE 6:**
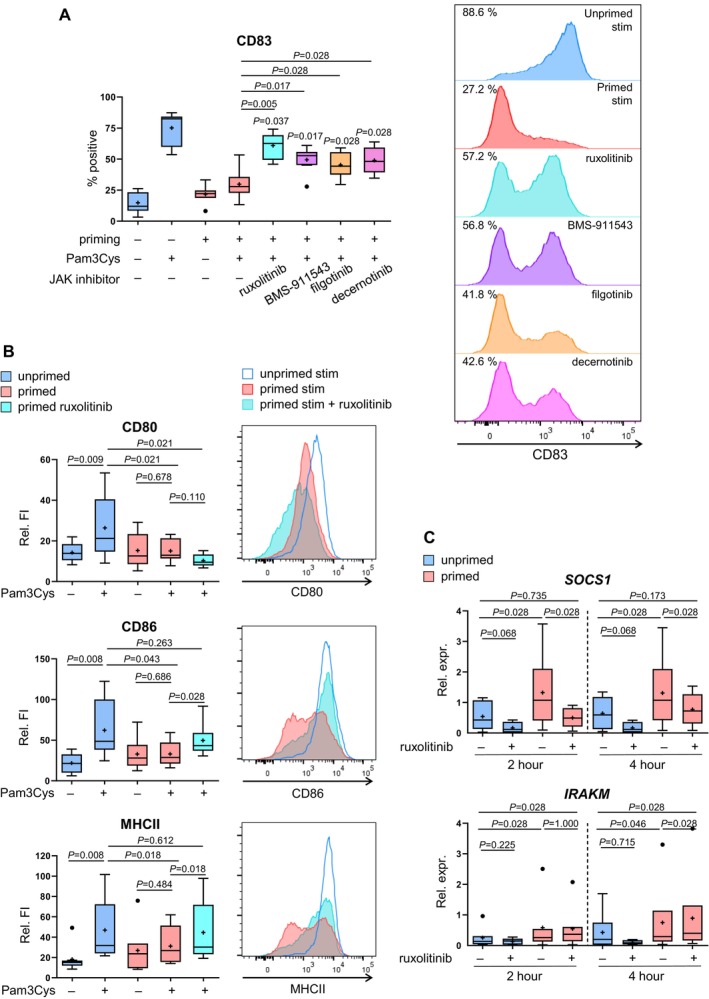
JAK Inhibitors can partly reverse priming induced changes in LC. (A) Pam3Cys‐primed and unprimed LC were treated with JAK inhibitors ruxolitinib (turquoise, *n* = 10), BMS‐911543 (purple, *n* = 6), filgotinib (orange, *n* = 8), or decernotinib (pink, *n* = 6) separately for 12 h, or left untreated (blue and red). Then cells were left unstimulated (−) or stimulated with 1 μg/mL Pam3Cys (+) for 24 h and stained with antibodies against CD83. B Primed and unprimed LC were treated with ruxolitinib (turquoise) or left untreated (blue and red) for 12 h and subsequently stimulated with 1 μg/mL Pam3Cys (+) or left unstimulated (−). CD80 (*n* = 9), CD86 (*n* = 9), MHCII (*n* = 8) or isotype control were analyzed on living CD1a + cells by flow cytometry. Boxplots show % of positive cells (A), and relative fluorescence intensity (Relative FI; B). The histograms show one representative experiment. (C) Pam3Cys‐primed and unprimed LC were treated with 1 μg/mL ruxolitinib for 2 h, 4 h, or left untreated. CD1a cells were enriched via magnetic separation. mRNA levels of *SOCS1* and *IRAKM* (*n* = 4–6) and *IRAKM* (*n* = 5–7) were quantified by qPCR. Boxplots show the relative expression fold change (Relative expression) after normalization, with the mean as “+” and outliers as “**•**”. Statistical significance was assessed using SPSS, applying the paired Wilcoxon signed rank test. *P*‐values are shown above the boxes, and in A above the boxplots indicates the difference from the second boxplot.

Ruxolitinib significantly increased CD83 expression in primed LC upon Pam3Cys stimulation, BMS‐911543 and decernotinib moderately raised CD83, whereas filgotinib had a slight effects (Figure 6A). Ruxolitinib also enhanced CD86 and MHC‐II expression in primed LC after Pam3Cys stimulation, but CD80 failed to be upregulated (Figure 6B). These findings suggest that JAK inhibitors, especially the JAK1/2 inhibitor, partly restore desensitized LC maturation.

Although the precise impact of JAK inhibitors on LC activation and immunogenicity remains unclear, JAK/STAT pathway activation was identified to elevate SOCS1 expression [41]. In light of this, we hypothesized that the JAK1/2 inhibitor‐driven restoration of maturation may involve the decrease of SOCS1 levels.

To explore ruxolitinib's repression potential, we assessed SOCS1 and IRAKM in ruxolitinib‐treated primed LC without subsequent Pam3Cys stimulation. SOCS1 expression significantly declined after 2 h and further decreased to baseline levels after 4 h in primed LC (Figure 6C). IRAKM, however, maintained high expression in primed cells.

Taken together, the JAK1/2 inhibitor partially restored desensitized LC maturation upon TLR2‐driven activation, potentially by suppressing the negative regulator SOCS1.

## 4 Discussion

We hypothesized that LC hypo‐responsiveness in AD skin toward TLR‐mediated activation [22] may be due to repeated TLR engagement in situ. As a cause, *S. aureus* , highly colonizing AD skin, may constantly trigger the immune cells. In this study, we explored the molecular aspects and the underlying mechanisms behind TLR2‐ related LC responsiveness. The in situ *S. aureus* exposure was mimicked by subjecting LC to prolonged and repetitive exposure to low doses of TLR2 ligands, referred to as priming, effectively emulating the dysfunction observed in AD. Our results demonstrated that primed LC exhibited impaired activation, diminished migration capacity, and altered cytokine profile upon TLR2‐induced activation and induction of AD‐related factors. We further identified SOCS1 and IRAKM induction as potential mechanistical contributors. Elevated SOCS1 levels in primed LC were downregulated by JAK inhibitors, resulting in restored maturation in response to TLR2‐mediated activation.

Firstly, we showed that the priming mimicking the LC exposure to *S. aureus* in AD is followed by impaired TLR2 responsiveness, as described previously for LC in AD skin [22]. Although CD80, CD86, and MHCII, which are directly involved in CD4 T cell interaction, were hardly affected by TLR2 engagement in primed LC, CD83 was upregulated to a small extent in our study. CD83 is intracellularly preformed in DC, and its localization outside and within the cells differs already in DC subtypes [42]. Though CD83 also has stabilizing abilities for MHCII and CD86 [43], other multiple functions in immune modulation and also wound healing have been proposed. Its stable surface expression in mature DC is contrary to its differentially regulated expression in macrophages depending on the stimulation, kinetics, and other yet unexplored factors [44, 45]. Additionally, CD83 in a soluble form can modify macrophage functions, which was not addressed in the current context [46]. It remains speculative if this small but significant change in primed LC after TLR stimulation has further functionally relevant effects, and future studies may shed light on the role of CD83 in LC biology in general and the context of AD.

Consistent with previous studies demonstrating cross‐tolerance between TLR2, TLR4, TLR3, and TLR5 [47, 48, 49], we also showed that desensitized LC occurred on both homologous TLR2 and heterologous TLR4. Interestingly, the priming with TLR4 ligand LPS was capable, but significant less effective in diminishing TLR2‐responsiveness of LC. Our results suggest that the colonizing bacteria composition of skin influences and regulates the responsiveness of LC and their functional outcome. In AD, these mechanisms may potentially elevate the risk of various types of infections [47, 49, 50].

The TLR2‐induced migration was impaired in primed cells compared to unprimed cells similar to previously described TLR2‐induced migration of AD skin LC compared to healthy skin LC [22]. This is in line with the lack of CCR7 expression in primed LC independently from the stimulation. However, AD skin LC showed a high spontaneous migratory activity, which was absent in the migration assay of the in vitro generated and primed LC we addressed in this study. Nontheless, the expression of CCR6 was diminished in primed LC independent of the stimulation, suggesting a reduced competence to interact with the epidermal compartment and thus a less fixed situation in the epidermis. Indeed, LC in AD lesional skin express lower CCR6 than healthy controls [51]. Thus, the spontaneous migratory behavior of LC in AD skin we observed previously, resulting in diminished LC numbers, might be explained by reduction of CCR6 as a result of constitutive exposure to *S. aureus* [22].

The primed LC exhibited a distinct cytokine profile upon TLR2‐driven activation. The reduced production of IL‐6, IL‐8, IL‐10, and IL‐23 by the hypo‐responsive LC upon TLR ligation is consistent with previous studies on tolerance and tolerogenic DC [36, 52], and in ex vivo skin of AD, TLR2‐induced IL‐6, and IL‐10 was reduced as well [22]. However, IL‐1β production was found to be decreased after TLR‐ or other stimulus‐induced hypo‐responsiveness in macrophages and moDC [53, 54], whereas IL‐18 exhibited increased production upon re‐stimulation [55, 56], in line with our results. We showed a significant increase in the release of both IL‐1β and IL‐18 following TLR2 stimulation in primed LC. This indicates a priming‐induced functional change of LC and strongly suggests the involvement of NLRP3 inflammasome. Interestingly, a role of the inflammasome in AD and its development is discussed in the NLRP1 gene and NLRP3 polymorphism have been associated with AD [57]. Our observation also aligns with the elevated levels of IL‐1β and IL‐18 found in the supernatant of AD skin, which are generally high [22]. In the epidermis, keratinocytes contribute to the overall high IL‐18 profile in AD. Additionally, IL‐1β and IL‐18 have been identified as pathogen‐associated alarmin cytokines that activate skin‐resident group 2 innate lymphoid cell (ILC2) and subsequently trigger Th2 cell‐mediated immune responses in AD [4, 58, 59, 60, 61, 62, 63, 64]. Moreover, IL‐1β is involved in *S. aureus* ‐driven regulation of lipids and thus skin barrier function in AD skin [65]. Thus, LC may contribute to these mechanisms of skin barrier disruption.

Although LC perform different functions during AD pathogenesis, they traditionally have not been considered as producers of IL‐1β and IL‐18. However, our results show that under certain conditions, including those mimicking the AD situation and under the influence of constitutive exposure to TLR2 ligands, LC are a robust source of those cytokines.

Additionally, TNF‐α production in ex vivo AD skin (data not shown) remained strongly responsive to TLR2 stimulation, which is consistent with our findings in primed LC. This is in contrast to studies on tolerogenic DC, where TNF‐α production was significantly suppressed upon re‐stimulation [54, 66]. Taken together, the discrepancies in high IL‐1β and IL‐18 levels and the unsuppressed TNF‐α production in response to TLR2‐driven activation between desensitized LC and known tolerogenic DC highlight the distinct features of these two cell states.

Our findings suggest that desensitized LC have impaired maturation and upregulation of costimulatory molecules CD80/CD86 and MHCII involved in T cells activation and diminished induced migratory capabilities, undermining their classical function as APC. Furthermore, these desensitized LC showed reduced production of cytokines associated with Th17 immune response, potentially attenuating LC capacity to effectively built control mechanisms of bacterial infections. However, the herein shown switch in the cytokine profile suggests that depending on the microbial environment the function of LC may switch to an IL‐1β/IL‐18‐mediated T2 immunity‐driving profile.

Mechanistically, primed LC showed an elevation of inhibitory molecules SOCS1 and IRAKM, whereas activating molecules of the TLR‐NF‐kB pathway were hardly affected. Though a trend of surface TLR2 reduction was observed in primed cells, the level alone did not influence the responsiveness of the LC, as shown by low‐expressing unprimed cells (Figure S2). A reduction of SOCS1 by JAK inhibitors, which are efficacious drugs for the therapy of AD, was accompanied by restored maturation of the cells upon TLR2‐engagement confirming that SOCS1 contributes to the functional change of the LC. In line with this, DC recurrently exposed to TLR ligands show SOCS1 induction, contributing to their poor response [67, 68, 69, 70]. Additionally, SOCS1 inhibits the release of TLR‐induced inflammatory cytokines such as IL‐6, TNFα, IFN‐γ, IL‐12, and IL‐10 [68, 69, 71] and regulates TLR ligand‐induced DC maturation. Interestingly, SOCS1 is highly expressed in the epidermis of chronic AD skin but not in non‐lesional skin [72]. Though SOCS1 is a negative regulator of the TLR signaling pathway and induced by several TLR ligands in DC, its transcriptional regulation by the TLR pathway remains controversial. TLR2‐mediated engagement activated STAT1 phosphorylation in murine RAW264.7 macrophages [73], which is known to induce SOCS1 expression, and TLR2 ligand LTA‐induced SOCS1 mRNA expression depend on MyD88 in peritoneal macrophages [74]. On the other hand, it has been identified that activation of the JAK–STAT pathway can induce the expression of SOCS1 by acting as transcription factors for STAT1 and STAT3, creating a negative feedback loop to regulate the signaling pathway [24, 25, 41]. Our results show that JAK inhibitors downregulate the elevated SOCS1 expression, thus indicating an indirect STAT‐dependent mechanism. DC show SOCS1 induction by TLR‐associated cytokines, such as type I and II interferons and IL‐6, via the JAK/STAT signaling pathway [25, 41, 75, 76, 77]. However, in our study, Pam3Cys did not induce IFN‐α or IFN‐γ in LC and blocking IL‐6 by blocking‐antibodies did not inhibit the development of LC into a desensitized state toward Pam3Cys stimulation (data not shown). In contrast, IRAKM was not affected by JAK inhibitors, which is a member of the IRAK family involved in activation of the TLR‐NF‐κB signaling pathway [38, 78]. Thus, the regulation of IRAKM in primed LC appears to follow a different mechanism than SOCS1 and appears to be independent from JAK/STAT‐signaling. It may explain why functional restoration of primed LC by JAK inhibitors occurs only partly. Furture studies, for example, in appropriate mouse models or JAK‐treated AD patients may unravel the full picture.

We provide evidence that SOCS1 induction by repetitive TLR2 stimulation results from an indirect induction via the JAK–STAT pathway. Nevertheless, the intricate cross‐talk mechanisms between the TLR2 and JAK–STAT signaling pathways in LC remain complex and require further investigation.

Taken together, we provide a suitable in vitro model for LC in AD, as seen previously in the AD skin, by the priming procedure mimicking constitutive *S. aureus* exposure. We could unravel a functional switch of the LC by diminishing the classical antigen‐presenting and T cell interacting factors and the directed migratory activity, but promoting the release of IL‐18 and IL‐1β, both of which can drive T2 immunity indirectly and are associated with AD. The underlying mechanism implies the inhibitory loop of TLR signaling, including SOCS1 and IRAKM, but not the acviatory molecules. We provide evidence that the SOCS1‐mediated regulation of TLR signaling is mediated indirectly by showing its JAK inhibitor sensitivity. The introduced model unravels important mechanistical aspects in AD, but also may serve as a model for the evaluation of therapeutical approaches in future.

## Author Contributions

Y.D. designed, performed and analyzed the experiments, and wrote the manuscript. N.H. designed and coordinated the project, supervised all experiments, analyzed the experiments, and wrote the manuscript. N.L. designed the priming approach. S.S. and S.B. performed experiments. J.O. provided the Buffy coat samples. T.B. designed and coordinated the project and edited the manuscript.

## Conflicts of Interest

Y.D. was supported by CK‐CARE, Chinese Scholarship Council (CSC) Scholarships and the Deutsche Forschungsgemeinschaft (DFG, German Research Foundation) under Germany's Excellence Strategy—EXC2151–390873048; N.H. and N.L. were supported by CK‐CARE. T.B. was speaker and/or consultant and/or Investigator for AbbVie, Affibody, Almirall, Amagma, AnaptysBio, AOBiom, Anergis, Apogee, Arena, Aristea, Artax, Asana Biosciences, ASLAN pharma, Astria, Attovia, BambusTx, Bayer Health, Belenos, BioVerSys, Böhringer‐Ingelheim, Bristol‐Myers Squibb, BYOME Labs, CellDex, Connect Pharma, Daichi‐Sanyko, Dermavant, DICE Therapeutics, Domain Therapeutics, DS Pharma, EQRx, EMD Serono, Galderma, Galapagos, Glenmark, GSK, Incyte, Innovaderm, Janssen, Kirin, Kymab, LEO, LG Chem, Lilly, L'Oréal, Mabylon, MSD, Medac, Micreos, Nektar, Nextech, Novartis, Numab, OM‐Pharma, Ornavi, Overtone, Pfizer, Pierre Fabre, Protagonist Tx, Q32bio, RAPT, Samsung Bioepis, Sanofi/Regeneron, TIRmed, UCB, Union Therapeutics, UPStream Bio, and YUHAN. He is founder and chairman of the board of the non‐profit biotech “Davos Biosciences AG” within the international Kühne‐Foundation and founder of the consulting firm “Bieber Dermatology Consulting”. J.O. has received research funding from Bayer, Biotest, CSL Behring, Octapharma, Pfizer, Swedish Orphan Biovitrum, and Takeda; consultancy, speakers bureau, honoraria, scientific advisory board, and travel expenses from Bayer, Biogen Idec, BioMarin, Biotest, Chugai, CSL Behring, Freeline, Grifols, LFB, Novo Nordisk, Octapharma, Pfizer, Roche, Sanofi, Spark Therapeutics, Swedish Orphan Biovitrum, and Takeda. All other authors have no conflicts of interest to declare.

## Supporting information


Data S1.

## Data Availability

The data that support the findings of this study are available on request from the corresponding author. The data are not publicly available due to privacy or ethical restrictions.
