# Supplementary material for: Langerhans Cell Modulation in Atopic Dermatitis Is TLR2/SOCS1‐Dependent and JAK Inhibitor‐Sensitive
Source: Allergy. 2025 Jul 9;80(9):2586–99. doi: 10.1111/all.16641 (PMC12444830; doi:10.1111/all.16641)
Supplement: Supplementary file 1 — Data S1. [file ALL-80-2586-s001.pdf]

Supp. Information

Reagents

LC medium was with, RPMI Medium 1640 (Life Technologies GmbH; Darmstadt, Germany), 10 µM 2-Mercaptoethanol, 10% heat-inactivated fetal bovine serum (both Sigma-Aldrich, St. Louis, USA), and 1% antibiotics/antimycotics (Gibco, Karlsruhe, Germany), GM-CSF (Sanofi-Aventis, NJ, USA), human myeloma IgE (Merck Millipore, Darmstadt, Germany), TNFα, stem cell factor (SCF), rhFlt3/Flk2 ligand (FLT-3L), and TGF-β (R&D Systems. Inc, Minneapolis, USA). TLR2 ligand Pam3Cys (EMC Microcollections, Tübingen, Germany), and TLR4 ligand LPS (InvivoGen, San Diego, USA). For flow cytometry, 7-amino-actinomycin D (7-AAD) (Sigma-Aldrich, St. Louis, USA), FITC-conjugated goat anti-mouse IgG and normal mouse serum (both Dianova, Hamburg, Germany). For the migration assay CCL19 (ImmunoTools. Friesoythe, Germany). cDNA were generated from cells by TRIzol® reagent (Life Technologies, Carlsbad, USA), DNAse-free kit (Thermo Fisher Scientific, Waltham, USA), and SuperScript™-II Reverse Transcriptase (Thermo Fisher Scientific, Waltham, MA, USA). For quantitative Real-time PCR, SYBR® Green (Bio-Rad Laboratories Inc., Hercules, USA), oligonucleotides (Life Technologies GmbH, Darmstadt, Germany) see Supp. Table S1. Taqman™ MicroRNA Assays, including hsa-miR-155-5p and RNU48 (Life Technologies GmbH, Darmstadt, Germany), see Supp. Table S2.

Flow cytometry

LC were gated for CD1a+ after dead cell exclusion by 7-AAD as described previously[1] and analyzed for the respective targets. Antibodies informations are given in Supp. Table S4. Samples were measured and analyzed by BD FACSCanto™ (Becton Dickinson, San Jose, Calif), and FlowJo 10.0.5 (Tree Star, Ashland, USA). The relative

fluorescence index (rFI) was calculated MFI(target) - MFI(isotype)/MFI(isotype), MFI = mean fluorescence intensity.

Tranwells migration experiment

Cell migration assessed by a 5.0µm transwell chamber (Corning, NY, USA). After 24h stimulation, 1× 10^6 cells resuspended in 100µL added to upper chamber, while 300 µL complete medium with 1 µg/mL of CC19L added to lower chamber. Incubated for 6h, migrated cells collected, counted, and average determined. CD1a expression percentage analyzed by flow cytometry.

mRNA and microRNA analyses

LC were sorted using CD1a beads via MACS® (Miltenyi Biotec, Bergisch Gladbach, Germany). cDNA from CD1a-enriched cells was synthesized and PCR performed by StepOne™ (Thermo Fisher Scientific, Waltham, USA). Data were analyzed with StepOne™ software using ΔΔCT method (relative quantity, RQ), normalized to β-actin mRNA level.

Cytokine production

Cytokines in LC culture supernatant after 24h of stimulation were analyzed with LEGENDplex™ (Biolegend, San Diego, CA, USA) according to the manufacturers instructions.

1. Herrmann N, Koch S, Leib N, et al. TLR2 down-regulates FcepsilonRI and its transcription factor PU.1 in human Langerhans cells. *Allergy.* 2013;68(5):621-628.

Supp. Table S1

Supp. Table S1 Sequences of the primers (5′– 3′) used for the qPCR analyses.

**Human target gene**	**Sequence forward primer**	**Sequence reverse primer**
ACTB	AGCGCGGCTACAGCTTCA	TCCTTAATGTCACGCACGATTT
CCR6	CCATTCTGGGCAGTGAGTCA	GCACGTGGCATTGCTGAA
CCR7	GCTGCGTCAACCCTTTCTTG	AAGAGATCGTTGCGGAACTTG
TLR1	TGTGCTGCCAATTGCTCATT	TTTTCCCCATAAGTCTCTCCTAAGAC
TLR2	CCAAGGAAGAATCCTCCAATCA	GCTGCCCTTGCAGATACCA
MyD88	TCACTGTCTGCGACTACACCAA	GGCAAGGCGAGTCCAGAAC
IRAK1	GCCCCTTTCCGTTTTGCT	ATCTTGAGCTCCTCCGAGAAGTT
IRAK2	GGAAACAGACGACGTTGACAATT	AGGGTGCCACACTCATGGA
IRAK4	CAGACTCTCTTGCTTGGATGGT	GCTGCACCCTGAGCAATCTT
TRAF6	CACCCCTGGAAAGCAAGTATG	ACTGCTTCTCGTAATGCCATCA
SOCS1	GCTGGCCCCTTCTGTAGGAT	CTGCTGTGGAGACTGCATTGTC
A20	CCATCCATGGACTGTGATTCTG	GAGCAGCTTGTTTTTCTGTCAATG
TOLLIP	CCTGATGCCAACAGTGTACCA	GCGGGCATCCCTGTGA
PI3K	TCTTAGGTCAGAAGTGCACATTCC	CCCCGGCAGTATGCTTCA
IRAKM	GCCTGGATTCATGTCTCTCATTT	AACAGAAGAGCTTGGCAGAGAAA
C-Fos	GGCGTTGTGAAGACCATGAC	TAACTGTTCCACCTTGCCCC
JAK1	CGAGATCCCCTTGAAAGACAAG	TGCACCGGCTTTCATAGAATC
JAK2	TGCTCCAGAATCACTGACAGAGA	ACCACTCCAAAGCTCCAAACA
JAK3	TGCCATCAACAAGCTCAAGACT	GCTGCGGCGGAGAACAT
TYK2	TTCTCTCTGCGTCGCTGTTG	CCGCATGATGATGAGATTGG
SOCS3	CCAGCCTGCGCCTCAA	CTTGCGCACTGCGTTCAC

Supp. Table S2

Supp. Table S2 Micro-RNA (miRNA) molecules used for transfection experiments.

miRNA precursor and inhibitor molecules were purchased from Life Technologies GmbH; Darmstadt, Germany.

Micro-RNA Name	miRBase Accession #	Species
Ambion® Pre-miR™ Precursor for hsa-miR-155-5p	MIMAT0000646	Homo sapiens
Ambion® Pre-miR™ miRNA Precursor Molecules - Negative Control # 1	synthetically derived miRNA	-
BLOCK-iT™ Alexa Fluor® Red Fluorescent Control (Ambion™)	synthetically derived miRNA	-

Supp. Table S3

Supp. Table S3 IC50 values of different JAK inhibitors

IC50, Half maximal inhibitory concentration.

Drug	JAK1	JAK2	JAK3	TYK2
**Filgotinib, nmol/L**	10	28	810	116
**BMS-911543, nmol/L**	385	1.1	82.5	71.5
**Decernotinib (VX-509), nmol/L**	11	13	2.5	11
**Ruxolitinib, nmol/L**	3.3	2.8	364	16.8
**Filgotinib, nmol/L**	10	28	810	116

Supp. Table s4

Supp. Table S4 Antibodies used for flow cytometry.

* mAb L243 against MHC class II were kindly provided by Dr. G. Moldenhauer (Heidelberg, Germany).

Antibody / conjugation	Immunogen	Clone	Species	Isotype	Manufacturer
**CD14-APC**	Human CD14	TÜK4	Mouse	IgG2a, κ	Miltenyi Biotec GmbH; Bergisch Gladbach, Germany
**CD1a-RD1 (T6-RD1)**	Human CD1a	SFCI19Thy1 A8	Mouse	IgG1	Beckman Coulter Inc.; Krefeld, Germany
**Goat anti-mouse IgG Fcγ –FITC**		polyclonal	Goat	IgG	Jackson ImmunoResearch Europe Ltd.; Suffolk, UK
**Langerin (CD207)**	Human CD207	4C7	Mouse	IgG2a	BioLegend, SanDiego, USA
**CD83**	Human CD83	HB15a	Mouse	IgG2b	Santa Cruz Biotechnology
**CD80**	Human CD80	L307.4	Mouse	IgG1	BD Pharmingen, Heidelberg, German
**CD86**	Human CD86	IT2.2	Mouse	IgG2b	BD Pharmingen, Heidelberg, German
**MHC-II**		L243	Mouse		provided*
**CCR6**	Human CD196/CCR6	11A9	Mouse	IgG1	BD Pharmingen, Heidelberg, German
**CCR7**	Human CCR7	150503	Mouse	IgG2a	R&D Systems Minneapolis, USA
**TLR2**	Human CCR7	1030A5.138	Mouse	IgG1	Immgenex; San Diego, USA
**IgG2a-APC**	isotype	S43.10	Mouse		Miltenyi Biotec GmbH; Bergisch Gladbach, Germany
**IgG1-RD1**	isotype	2T8-2F5	Human		Beckman Coulter Inc.; Krefeld, Germany
**IgG2b**	isotype	MOPC-141	Mouse		Sigma-Aldrich, Taufkirchen, Germany
**IgG2a**	isotype	UPC-10	Mouse		Sigma-Aldrich, Taufkirchen, Germany

Supp. Figure S1

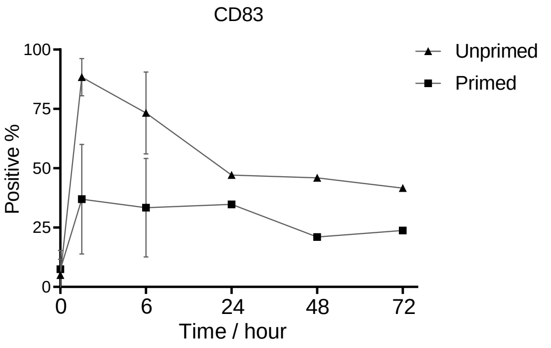

Supp. Figure S1 Duration of priming-induced desensitization in LC
LC were generated in vitro as previously described and subjected to either Primed or Unprimed conditions. Cells were then either unstimulated (-) or stimulated (+) with Pam3Cys (1 µg/mL) for 6 hours(n=10), 24 hours(n=11), 48 hours(n=2), and 72 hours(n=2). Cells were labeled with antibodies targeting CD83, or an isotype control. Gating was performed using CD14 and CD1a markers, and dead cells were excluded using 7-AAD staining. Flow cytometry was employed for analysis. Results are displayed as the percentage of positive cells within the population (Positive %), presented as mean ± SEM.

Supp. Figure S2

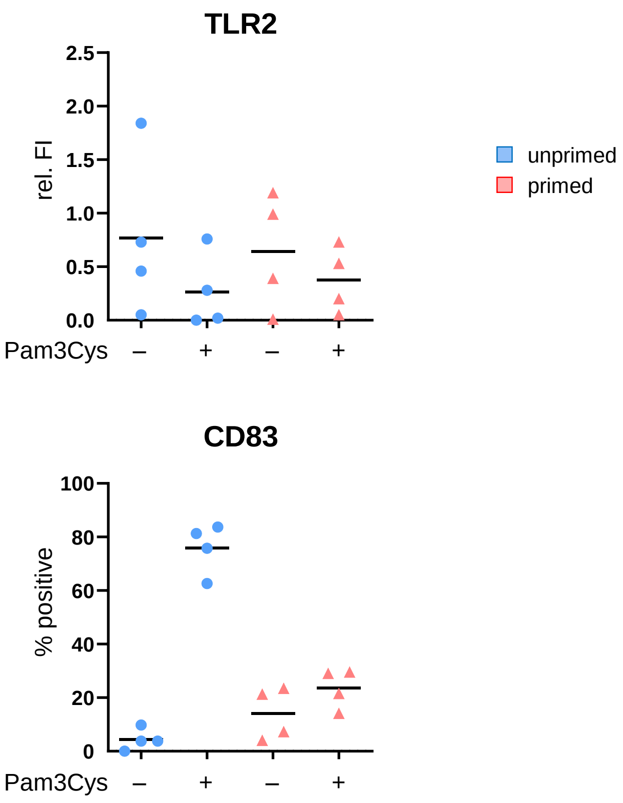

Supp. Figure S2 TLR2 surface expression is not associated with the activation capacity of LC.
LC were generated *in vitro* as previously described and subjected to either primed (red triangels) or unprimed (plue circles) conditions. Cells were stimulated with Pam3Cys for 24h. Cells were labeled with antibodies targeting TLR2 (upper plot) and CD83 (lower plot) or isotype control. Gating was performed using CD14 and CD1a markers, and dead cells were excluded using 7-AAD staining. Flow cytometry was employed for analysis. Results of 4 individual experiments are presented. The mean is given as black line.

Supp. Figure S3

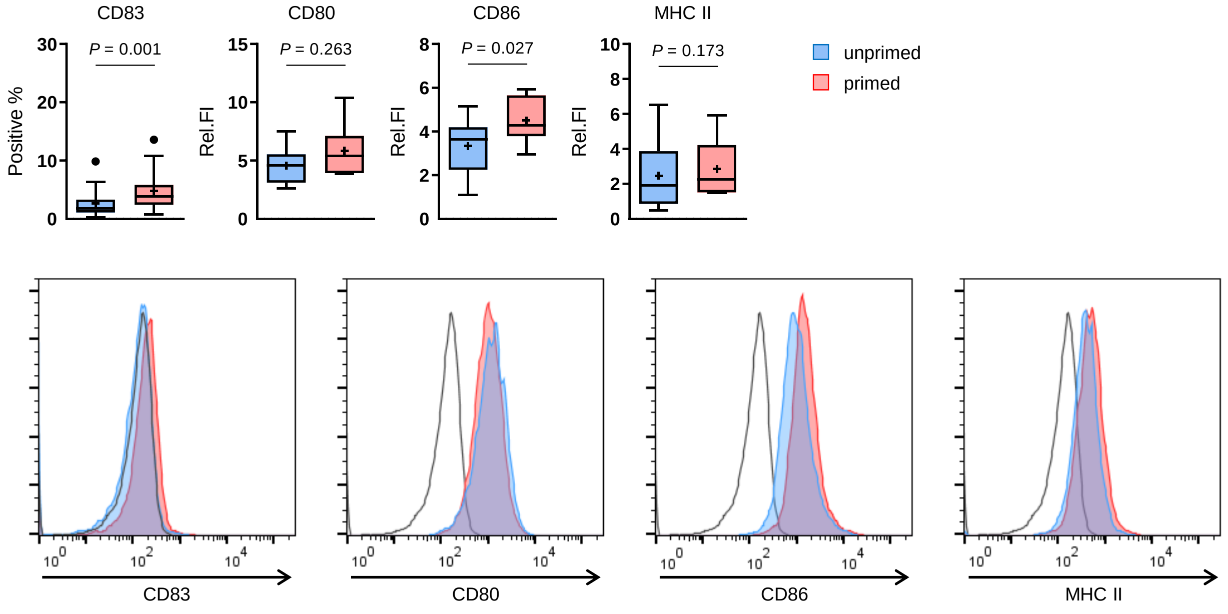

Supp. Figure S3 Maturation Molecules CD83, CD80, CD86, and MHC-II in Primed LC
LC were generated in vitro as previously described and subjected to either Primed or Unprimed conditions. Analysis after 24 hours of priming treatment in absent of Pam3Cys.
Cells were labeled with antibodies targeting CD83(n=16), CD80(n=7), CD86(n=7), MHC II(n=7), or isotype control. Gating was performed using CD14 and CD1a markers, and dead cells were excluded using 7-AAD staining. Flow cytometry was employed for analysis. Results are presented as the percentage of positive cells within the population (Positive %) and as mean of relative fluorescence intensity (Rel.FI).
The histogram represents one representative experiment depicting, isotype(empty), unprimed cells (blue) and primed cells (red). Results are presented as boxplots, with the mean indicated by "+", and outliers shown as "●". Statistical significance was assessed using SPSS and the Wilcoxon Signed Ranks Test. P-values are shown about the graph.
